# Supplementary material for: New Poplar-Derived Biocomposites via Single-Step Thermoforming Assisted by Phosphoric Acid Pretreatment
Source: Polymers (Basel). 2022 Sep 2;14(17):3636. doi: 10.3390/polym14173636 (PMC9460407; doi:10.3390/polym14173636)
Supplement: Supplementary file 1 [file polymers-14-03636-s001.zip › polymers-1846561-supplementary.pdf]

Supplementary Materials for

New poplar-derived biocomposites via single-step thermoforming assisted by phosphoric acid pretreatment

This file includes:

Table S1

Figs. S1

Table S1. The methods of pretreatment

NO.	Name	Phosphoric acid concentration	Time	Method/parameter
**1**	WF	—	—	—
**2**	WF (PA)	5%	3 h	—
**3**	WF(PA/M)	5%	3 h	Microwave process 400 W<br>80 ℃, 20 min
**4**	WF(PA/B)	6%	—	Ball milling 300 r/min、<br>150 ℃<br>2 h, solid-to-liquid ratio: 1:6

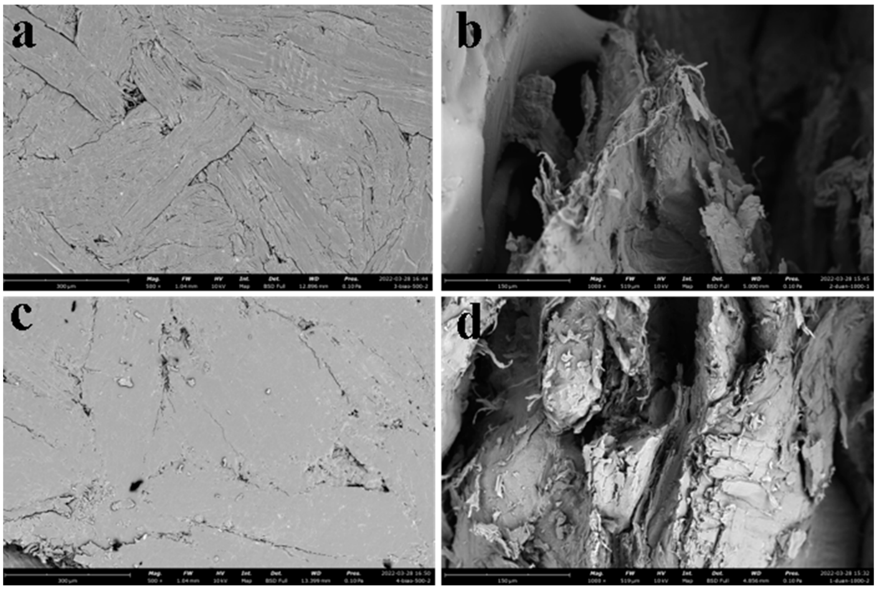

Fig. S1. (a) Microscopic image of the surface of WF (PA). **(b)** Microscopic images of the cross-section of WF (PA). **(c)** Microscopic image of the surface of WF (PA/M). **(d)** Microscopic images of the cross-section of WF (PA/M).
